# Clear cell sarcoma of the right lumbar region: A case report and review of the literature

**DOI:** 10.3892/ol.2014.2372

**Published:** 2014-07-22

**Authors:** XUE-LI YANG, SAN-JUN LU, JIE XUE, YAN-FEN WU, JUN-LING SHI

**Affiliations:** Department of Pathology, The First Hospital of Handan City, Handan, Hebei 056002, P.R. China

**Keywords:** clear cell sarcoma of soft tissues, S-100, MITF, melanin

## Abstract

Clear cell sarcoma of soft tissues is a very rare, malignant soft tissue tumor that usually arises in the extremities, with a predilection for the lower limbs. This report presents a 45-year-old male with a painless mass in the right lumbar region for one year. Magnetic resonance imaging showed a 3.6×3.2×1.5-cm soft tissue mass of the right lumbar region. The tumoral cells had pleomorphic nuclei and large amounts of clear cytoplasm, and a proportion of the cells contained melanin. Immunohistochemical analysis was performed, which identified that the cells were positive for S-100, MITF and HMB-45 tumor markers. The patient underwent a postoperative chemotherapy protocol and had no local recurrences at one year post-surgery.

## Introduction

Clear cell sarcoma of soft tissues (CCSST) was first described by Enzinger in 1965 (1). CCSST is a very rare, malignant soft tissue tumor and is often referred to as ‘malignant melanoma of soft tissues’ due to the histologic similarities and lack of observable pigmentation often seen in cutaneous melanoma. Clinically, the majority of cases present as a slowly progressive mass with a predilection for young females (2). Patients with CCSST have a variable unpredictable prognosis (3). The condition presents as a soft tissue mass common to the tendons, and aponeurosis is observed in the lower extremities and rarely presents in the trunk (2). The present study reports an unusual case of CCSST in the right lumbar region. To the best of our knowledge, this is the first case report that has been documented in the English literature worldwide regarding CCSST in the right lumbar region. Written informed consent was obtained from the patient.

## Case report

A 45-year-old male presented to our department with a painless mass in the right lumbar region of one year. The patient related that the mass had remained stable for 11 months, with no pain or increase in size. One month prior to presenting at the hospital, the patient reported that the mass had started to enlarge, elicit pain and affect the general wellbeing of the patient. The patient did not have personal or familiar history of cutaneous malignancy. Magnetic resonance imaging identified a 3.6×3.2×1.5-cm soft tissue mass of the right lumbar region.

Physical examination revealed a subcutaneous irregular firm mass in the right lumbar region region measuring 3.5×3.0 cm. There were no signs of superficial skin inflammation and clinical examination did not reveal other relevant cutaneous lesions.

**Figure 1:**
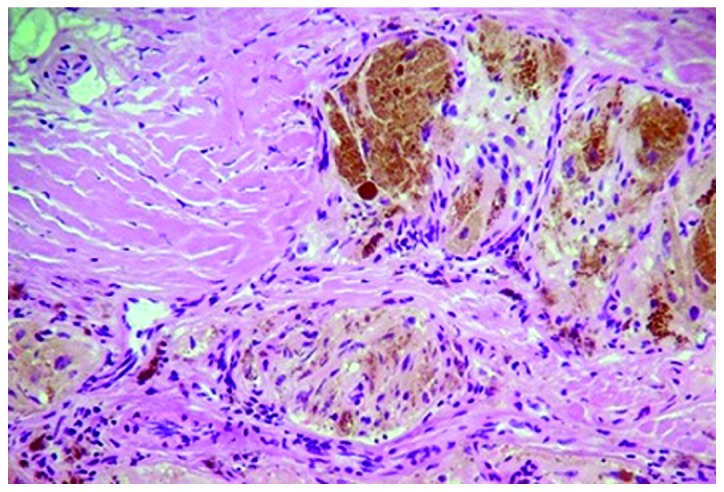
Tumoral cells were arranged in sheets and small nests, separated by connective tissue septa. Hematoxylin and eosin stain; magnification, ×100.

**Figure 2:**
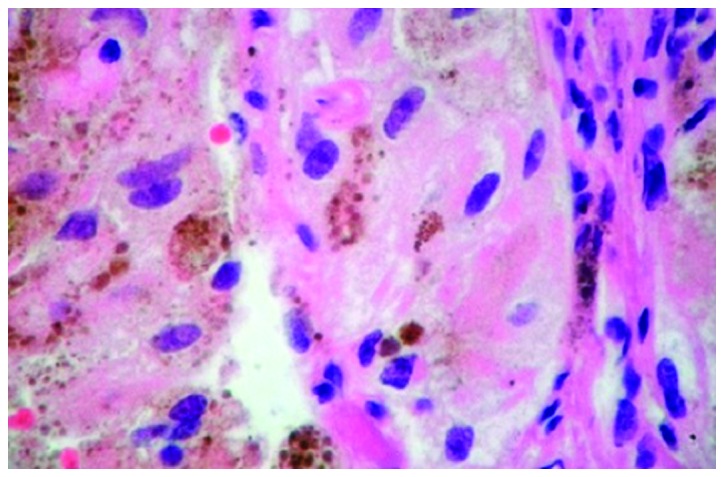
Tumoral cells exhibited pleomorphic nuclei and large amounts of clear cytoplasm. The spindle-shaped cells had weakly stained eosinophilic cytoplasm. Patchy melanin expression was observed. Hematoxylin and eosin stain; magnification, ×400.

**Figure 3:**
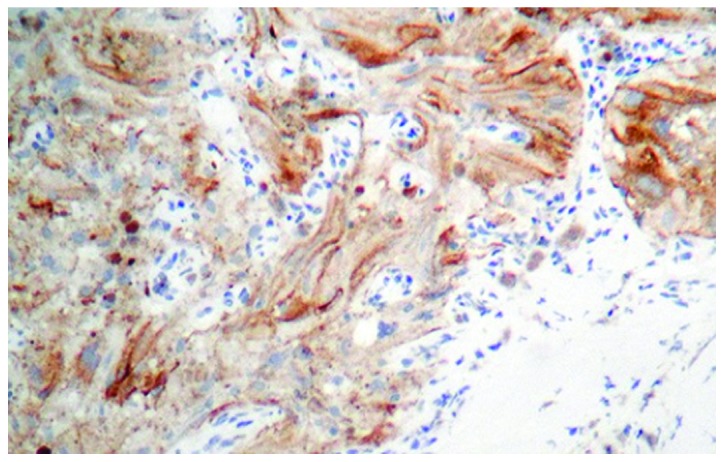
Immunohistochemical examination showed positive expression of S-100 tumor marker. Magnification, ×100.

**Figure 4:**
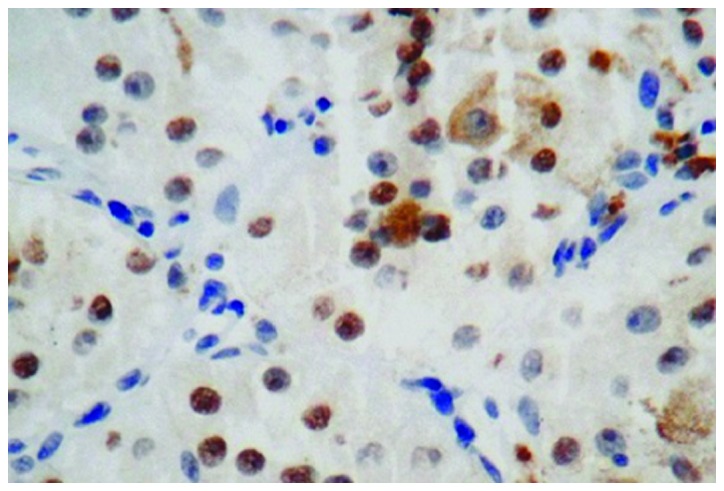
Immunohistochemical examination showed positive expression of MITF tumor marker. Magnification, ×200.

An open biopsy was performed and the gross specimen measured 3.5×3×1.2 cm. The gross specimen was described as a gray-white, homogenous, rubbery tissue with no attachment to the skin. Tumoral cells were arranged in sheets or small nests, and were separated by connective tissue septa (Fig. 1). The cells had pleomorphic nuclei and large amounts of clear cytoplasm, whereas the spindle-shaped cells had palely stained eosinophilic cytoplasm. Patchy melanin expression was observed (Fig. 2). Mitoses were moderately numerous and there was no evidence of necrosis or hemorrhage. Immunohistochemical analysis indicated positive staining for tumor markers S-100 (Fig. 3), MITF (Fig. 4) and HMB-45, and negative staining for CD10, CD68, actin, desmin and AE1/AE3 antigens (data not shown). The pathological findings were compatible with CCSST.

The patient was started on a postoperative chemotherapy protocol. Radiotherapy was not administered, as requested by the patient. The patient had no local recurrences at the one year post-surgery follow-up and there were no complications from the chemotherapy treatment.

## Discussion

CCSST is currently a distinct entity classified, by the World Health Organization, as soft tissue and bone tumors (4). CCSST is a rare tumor accounting for only a small percentage of soft tissue tumors. CCSST is an aggressive soft tissue tumor with a long period from the first symptom to diagnosis. The median age of patients at diagnosis is 33 years (5). Clinically, CCSST is most often present in young adults, with a slight female bias.

Pain above the tumor site may be experienced in 33–50% of all the patients that suffer from CCSST. Primary CCSST usually arises in deeper soft tissues, bound to an adjacent tendon or aponeurosis (6). Frequently, it arises in the extremities with a predilection for the lower limbs (78–97%). The foot and ankle are the most common sites of tumor appearance, accounting for 33–65% of all cases. The next most common sites are the knee, thigh, hand, forearm, elbow and shoulder, in descending order of frequency; tumors rarely arise in the head, neck or trunk (7–10). Primary CCSST overlying the right lumbar region, as in the present case, are uncommon.

Histologically, CCSST is composed of round and/or fusiform cells that are arranged in nests and separated by fibrocollagenous tissue. The cells have pleomorphic nuclei and large amounts of clear cytoplasm, while the spindle-shaped cells exhibit palely stained eosinophilic cytoplasm (5).

A small number of giant cells, with >12–15 nuclei, may be observed (11), but there were no wreath-like giant cells in the presented case. Melanosomes may be present, as detected at the ultrastructural level (11). In the present case, some cells were detected that contained melanin.

There are no specific immunoreactive markers used to delineate clear cell sarcoma. S-100 and HMB-45 are often used to differentiate clear cell sarcoma from epithelial tumors and synovial sarcoma, and faint keratin immunoreactivity has been observed in clear cell sarcoma (5). More recently, molecular genetic characterization of clear cell sarcoma has been shown to be specific for t(12;22) chromosomal translocation, which is typically not present in cutaneous malignant melanoma (MM) (12,13). This translocation has not been observed in either cutaneous or uveal MM or malignant peripheral nerve sheath tumors (MPNSTs+) (14,15). A chromosomal study was not performed in the present case due to practical constraints.

An important differential diagnosis is metastatic MM. A possible source of a primary lesion must first be excluded before making a diagnosis of CCSST (12). In contrast to MM, CCSST is situated in deep tissues and is generally located in non-pigmented areas.

Surgical resection, adjuvant radio- or chemotherapy or a combination of these three treatments has no reported significant advantage of one therapy over another (16), although a report has suggested that metastatic tumors exhibit almost no response to systemic chemotherapy (17). The described patient underwent a surgical excision and chemotherapy, and was healthy at the one-year follow-up.

Patients with CCSST have a variable unpredictable course of the disease. Lymph node metastasis has been reported in a high number of cases (12). The other sites of metastases often include the lungs, skin, bones, liver, heart and brain. Lymph node metastasis has a worse survival rate (12) and it has been hypothesized that the size of the tumor can define a better or worse prognosis (16). Other clinical and pathological factors have no significant association to survival or distant metastasis when the tumor size is >5 cm. In a study by Deenik *et al* (12), patients with a tumor size <2 cm had an improved survival. Preoperative duration of symptoms, mitotic index or vascular invasion may not predict survival in these patients.
